# Enhanced Production of Anthraquinones and Phenolic Compounds and Biological Activities in the Cell Suspension Cultures of *Polygonum multiflorum*

**DOI:** 10.3390/ijms17111912

**Published:** 2016-11-16

**Authors:** Muthu Thiruvengadam, Kaliyaperumal Rekha, Govindasamy Rajakumar, Taek-Jun Lee, Seung-Hyun Kim, Ill-Min Chung

**Affiliations:** Department of Applied Bioscience, College of Life and Environmental Sciences, Konkuk University, Seoul 143 701, Korea; muthu@konkuk.ac.kr (M.T.); krekhathiruvengadam@gmail.com (K.R.); govindr@konkuk.ac.kr (G.R.); Itjnazi@konkuk.ac.kr (T.-J.L.); kshkim@konkuk.ac.kr (S.-H.K.)

**Keywords:** anticancer activity, antimicrobial activity, cell suspension culture, emodin, physcion, phenolic compounds, *Polygonum multiflorum*

## Abstract

Anthraquinones (AQs) and phenolic compounds are important phytochemicals that are biosynthesized in cell suspension cultures of *Polygonum multiflorum*. We wanted to optimize the effects of plant growth regulators (PGRs), media, sucrose, l-glutamine, jasmonic acid (JA), and salicylic acid (SA) for the production of phytochemicals and biomass accumulation in a cell suspension culture of *P. multiflorum*. The medium containing Murashige and Skoog (MS) salts and 4% sucrose supplemented with 1 mg/L 2,4-dichlorophenoxyacetic acid, 0.5 mg/L thidiazuron, and 100 µM l-glutamine at 28 days of cell suspension culture was suitable for biomass accumulation and AQ production. Maximum biomass accumulation (12.5 and 12.35 g fresh mass (FM); 3 and 2.93 g dry mass (DM)) and AQ production (emodin 295.20 and 282 mg/g DM; physcion 421.55 and 410.25 mg/g DM) were observed using 100 µM JA and SA, respectively. JA- and SA-elicited cell cultures showed several-fold higher biomass accumulation and AQ production than the control cell cultures. Furthermore, the cell suspension cultures effectively produced 23 phenolic compounds, such as flavonols and hydroxycinnamic and hydroxybenzoic acid derivatives. PGR-, JA-, and SA-elicited cell cultures produced a higher amount of AQs and phenolic compounds. Because of these metabolic changes, the antioxidant, antimicrobial, and anticancer activities were high in the PGR-, JA-, and SA-elicited cell cultures. The results showed that the elicitors (JA and SA) induced the enhancement of biomass accumulation and phytochemical (AQs and phenolic compounds) production as well as biological activities in the cell suspension cultures of *P. multiflorum*. This optimized protocol can be developed for large-scale biomass accumulation and production of phytochemicals (AQs and phenolic compounds) from cell suspension cultures, and the phytochemicals can be used for various biological activities.

## 1. Introduction

*Polygonum multiflorum* Thunb. is a member of the family Polygonaceae and a traditional Chinese medicinal herb. It has been used in the preparation of herbal medicines in China, Japan, Korea, and Taiwan, as it has important pharmacological functions [1]. *P. multiflorum* possesses various biological activities, such as anticancer, anti-HIV, antimicrobial, and antioxidative activities, and it is used to blacken hair and treat hair loss and baldness [2]. Previous phytochemical studies have demonstrated that *P. multiflorum* has mainly two types of active components, anthraquinones (AQs) and stilbenes, as well as other compounds such as phenolics, flavonoids, tannins, and phospholipids [3]. The AQ derivatives of *P. multiflorum* have antimicrobial, anticancer, and antioxidant activities. Moreover, these metabolites are used as a dye in textile and food industries [4]. The biological effects of emodin include anticancer, antimutation, antimicrobial, and anti-inflammatory activities and prokinetic actions on gastrointestinal smooth muscles [2]. Phenolic compounds are a large group of secondary metabolites extensively distributed in plants, and they can be divided into two main subgroups: flavonoids and phenolic acids. Previous studies have suggested that polyphenols may play a vital role in preventing obesity, coronary heart disease, colon cancer, and gastrointestinal disorders, and they can also reduce the risk of diabetes [5]. Harvesting from the root cannot meet the growing demand for AQs and phenolic compounds for medical and commercial uses, and it also raises serious ecological concerns [1]. Therefore, isolation and purification of AQs and phenolic compounds have attracted much attention for large-scale production.

The production of secondary metabolites by field-grown intact plants has various disadvantages, such as low yields, slow growth cycles, fluctuations in quantity due to unfavorable environmental conditions, infestation, and disease. Plant tissue or cell culture technology, an attractive alternative system for uniform phytochemical synthesis, can continuously offer high-value medicines, foods, and healthy ingredients, independent of geographical, climatic, or environmental variations and constraints [6]. Various important chemical compounds such as AQs, alkaloids, saponins, carotenoids, anthocyanins, and polyphenols are synthesized and accumulated in cultured plant cells or tissues [7,8]. Elicitation is also an effective method for improving secondary metabolite production in cell and organ culture [7,9,10]. Jasmonic acid (JA) and salicylic acid (SA) are plant hormones and signaling molecules that play a major role in abiotic or biotic stress tolerance [11]. JA and SA have previously been used as elicitors in tissue or cell culture to enhance the production of secondary metabolites [12,13]. Elicitation of JA and SA increased the content of 2,3,5,4′-tetrahydroxystilbene-2-*O*-β-d-glycoside in the suspension cells of *P. multiflorum* [14]. We optimized the parameters for cell suspension culture established from root explants and elicitation of cultures for enhanced production of biomass and phytochemicals (AQs and phenolic compounds) of *P. multiflorum*. We have evaluated 23 phenolic compounds and biological activities (antioxidant, antimicrobial, and anticancer activities) in the cell suspension cultures of *P. multiflorum*. This study is the first report of using elicitors (JA and SA) for inducing the production of phytochemicals (AQs and phenolic compounds) and enhancing biological activities in the cell suspension cultures of *P. multiflorum*.

## 2. Results and Discussion

### 2.1. Establishment of Callus Culture

Important phytochemicals such as AQs [12,15] and phenolic compounds [16] are produced from callus and cell suspension cultures of several medicinal plants. We selected the root as the explant for callus culture, since many principal components are biosynthesized in the roots of *P. multiflorum*. The root explants consistently produced friable callus and were used for the cell suspension cultures of *Withania somnifera* [17]. The callus cultures were successfully established from the root explants obtained from in vitro seedlings of *P. multiflorum*. Previous studies have demonstrated that auxins and cytokinins directly influence the biosynthesis of secondary metabolites in plant cell or tissue culture [18,19]. Appropriate concentration of the medium is a critical determinant for controlling callus growth and metabolite production. We investigated different cytokinins and auxins, alone or in combination, in solid MS media. For three weeks, white and friable calli were obtained on MS solidified medium containing 3% sucrose supplemented with 1 mg/L 2,4-D, 0.5 mg/L BAP, and 0.5 mg/L KN.

### 2.2. Effects of Different Concentration of Auxins and Cytokinins on Biomass Accumulation and AQ Production in the Cell Suspension Culture

**Figure 1:**
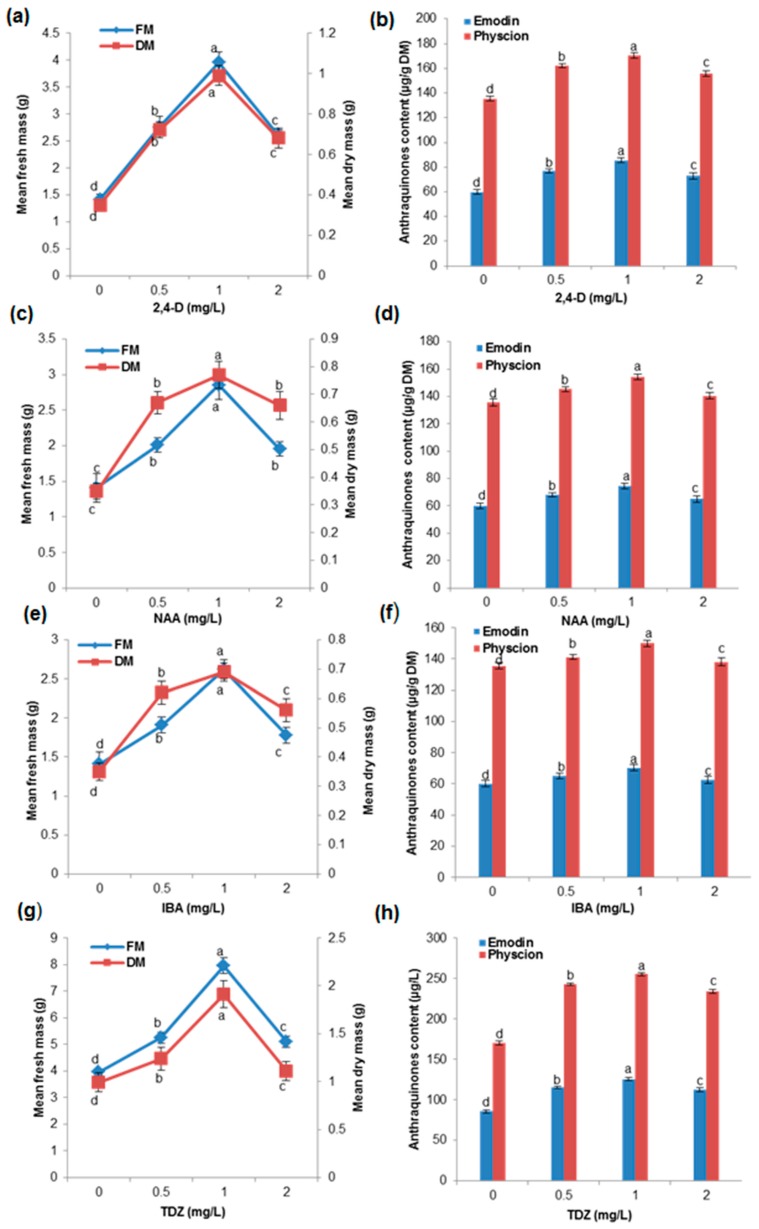
Effects of different concentrations of auxins and cytokinins in combination with biomass accumulation and anthraquinone (emodin and physcion) production in cell suspension cultures of *P. multiflorum*; (**a**,**b**) 2,4-dichlorophenoxyacetic acid (2,4-D); (**c**,**d**) naphthaleneacetic acid (NAA); (**e**,**f**) indole-3-butyric acid (IBA); (**g**,**h**) 2,4-D 1.0 mg/L with thidiazuron (TDZ). Means ± standard deviation of three replicates followed by the same letters are not significantly different according to Duncan’s multiple range test (DMRT) at *p* ≤ 0.05. DM: dry mass; FM: fresh mass.

Different concentrations of auxins were tested, and among them, 2,4-D at 1 mg/L in liquid MS medium resulted in maximum biomass accumulation (3.95 g FM and 0.99 g DM) and AQ production (emodin 85.55 mg/g DM and physcion 170.44 mg/g DM) (Figure 1a,b). NAA and IBA produced less biomass accumulation and AQ (emodin and physcion) production (Figure 1c–f). We selected 2,4-D (1 mg/L) as the best auxin for our study as it exhibits a stimulatory effect on the accumulation of secondary metabolites in the cell suspension cultures of several medicinal plants [20,21]. Moreover, 2,4-D was essential for anthocyanin production and biomass accumulation in the cell suspension cultures of *Cleome rosea* [22]. Exogenously applied cytokinins along with auxins altered cell division, promoted the proliferation of plant cell suspension culture, and enhanced secondary metabolites in *W. somnifera* [17]. The addition of cytokinins (BAP, KN, and TDZ) has been shown to improve the production of secondary metabolites in the cell suspension culture of *Panax ginseng*, *Daucus carota*, and *Artemisia absinthium* [20,21,23]. In our study, 1 mg/L 2,4-D along with 0.5 mg/L TDZ caused maximum biomass accumulation (7.95 g FM and 1.91 g DM) and AQ production (emodin 125.25 mg/g DM and physcion 255 mg/g DM) (Figure 1g,h). This proves that auxins, cytokinins, and their interactions significantly manipulate growth and metabolic activities in the cell suspension culture of *P. multiflorum*.

### 2.3. Effects of Growth Kinetics on Biomass Accumulation and AQ Production in the Cell Suspension Culture

**Figure 2:**
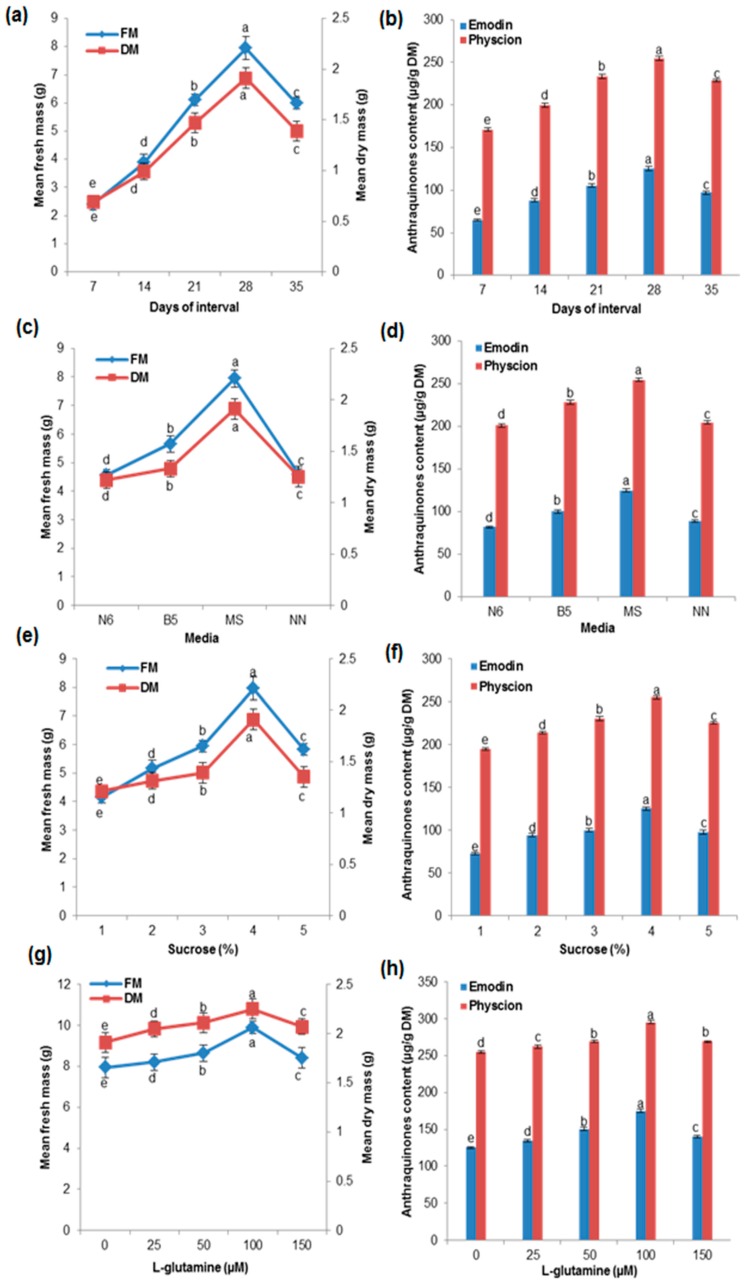
Effects of various media and concentration of sucrose, l-glutamine and growth kinetics of biomass accumulation and anthraquinone (emodin and physcion) production in cell suspension cultures of *P. multiflorum*: (**a**,**b**) Growth kinetics; (**c**,**d**) media; (**e**,**f**) sucrose; (**g**,**h**) l-glutamine. Means ± standard deviation of three replicates followed by the same letters are not significantly different according to DMRT at *p* ≤ 0.05.

The time course for biomass (FM and DM) accumulation and production of AQs (emodin and physcion) are shown in Figure 2a,b. The maximum accumulation of biomass (7.95 g FM and 1.91 g DM) and AQ production (emodin 125.25 mg/g DM and physcion 255.00 mg/g DM) were recorded at 28 days. Consistently, the 28-day old cell suspension culture showed a higher amount of metabolite production and biomass accumulation in *W. somnifera* [24] and *A. absinthium* [23]. The cell growth in the suspension culture exhibited two phases: the exponential phase (days 7–28) and decline phase (days 29–40). The plant cells needed to adjust to the new environment in the lag phase and accumulated the maximum biomass and secondary metabolites in the exponential phase [25].

### 2.4. Effects of Various Media and Different Concentrations of Sucrose and Glutamine on Biomass Accumulation and AQ Production in the Cell Suspension Culture

The concentration of nutrients in the medium is a major factor for controlling the growth of cells and accumulation of secondary metabolites [24]. In the present study, different media like MS, SH, B5, and N6 were used for biomass accumulation and production of AQs, and the results showed that MS medium was better than the other media for both biomass accumulation and AQ production (Figure 2c,d). The highest accumulation of biomass (7.95 g FM and 1.91 g DM) and AQ production (emodin 125.25 mg/g DM and physcion 255.00 mg/g DM) were recorded in MS medium. Consistently, MS medium is suitable for biomass accumulation and secondary metabolite production in cell suspension cultures [14,17,23,24]. Sucrose has been documented to act as a primary energy source for signaling molecules, and it affects the growth development and biosynthesis of secondary metabolites in cultured cells [26]. We studied the effects of sucrose concentration (1%–5%) in MS medium on the increase in biomass accumulation and AQ production (Figure 2e,f). In the present study, 4% sucrose concentration was found suitable for biomass accumulation (7.95 g FM and 1.91 g DM) and AQ production (emodin 125.25 mg/g DM and physcion 255.00 mg/g DM). Similarly, 4% sucrose resulted in higher biomass accumulation and secondary metabolite production [23,24]. Lower sucrose concentration cannot provide adequate energy and therefore may not be able to act as a building block, which is consistent with the findings of our study. However, higher sucrose concentrations have an adverse effect on growing cells [23]. Biomass accumulation and AQ production varied with different concentrations of l-glutamine (Figure 2g,h). Maximum biomass accumulation (9.90 g FM and 2.25 g DM) and AQ production (emodin 175.00 mg/g DM and physcion 295.50 mg/g DM) were observed in liquid MS medium containing 4% sucrose supplemented with 100 µM l-glutamine, 1 mg/L 2,4-D, and 0.5 mg/L TDZ. In accordance with our reports, the maximum biomass accumulation and metabolite production of *Artemisia absinthium* were in the MS medium with PGRs and l-glutamine [23]. The presence of l-glutamine, which is relatively non-toxic, permitted cells to sustain a high growth rate for a longer period [27]. The present study showed that l-glutamine interacted with the PGRs and promoted cell division and biosynthesis of AQs in the cell suspension culture.

### 2.5. Effects of Elicitors (JA and SA) on Biomass Accumulation and AQ Production in the Cell Suspension Culture

**Figure 3:**
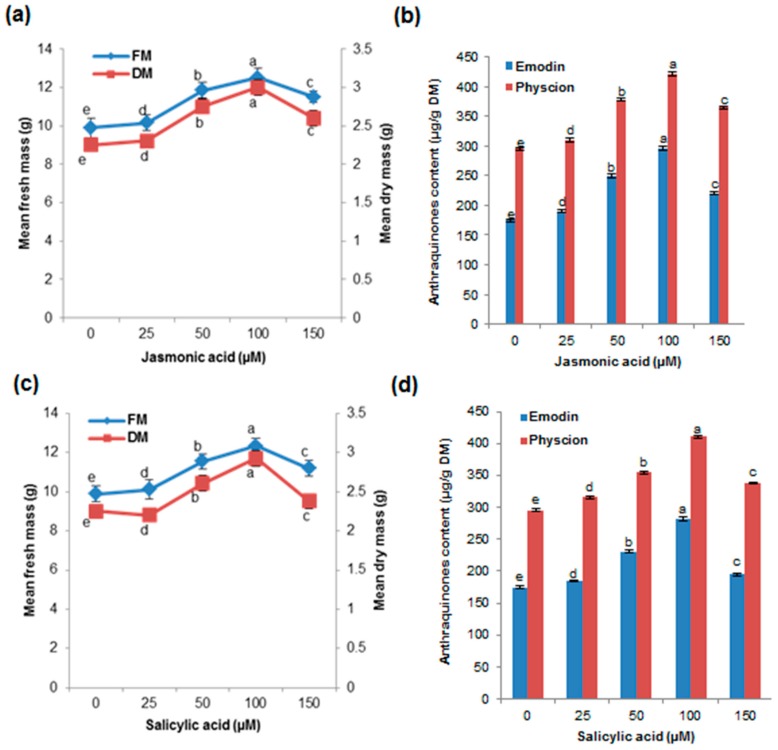
Effects of jasmonic acid (JA) and salicylic acid (SA) on biomass accumulation and anthraquinone (emodin and physcion) production in cell suspension cultures of *P. multiflorum*: (**a**,**b**) JA; (**c**,**d**) SA. Means ± standard deviation of three replicates followed by the same letters are not significantly different according to DMRT at *p* ≤ 0.05.

Two elicitors (JA and SA) were tested with respect to biomass accumulation and AQ production in the cell suspension culture of *P. multiflorum*. Addition of the elicitors significantly increased the biomass (FM and DM) and AQ (emodin and physcion) production (Figure 3a–d). High levels of biomass accumulation (12.50 g FM and 3 g DM) and AQ production (emodin 295.20 mg/g DM and physcion 421.55 mg/g DM) were observed in the medium with 4% sucrose and PGRs (l-glutamine 100 µM, 2,4-D 1 mg/L, TDZ 0.5 mg/L) and supplemented with JA (100 µM) in the 28-day cell suspension culture of *P. multiflorum* (Figure 3a,b).

In *Morinda citrifolia*, 100 µM methyl jasmonate (MeJA) elicited a four-fold higher AQ yield than that in the control, which is consistent with the findings of our study [12]. Similar to our results, 100 µM MeJA and SA significantly increased the content of 2,3,5,4′-tetrahydroxystilbene-2-O-β-d-glycoside in cell suspension cultures of *P. multiflorum* [14]. SA (100 µM) elicitation induced biomass accumulation (12.35 g FM and 2.93 g DM) and AQ production (emodin 282.00 mg/g DM and physcion 410.25 mg/g DM) (Figure 3c,d). Consistently, SA elicitation increased the biomass production and anthocyanin accumulation in the callus and cell suspension cultures of *Vitis vinifera* [28]. The synergistic or additive effect of JA or SA on cell suspension growth and secondary metabolite production has been well established in several plant species [10,12,14,29]. The results suggest the use of elicitation (JA or SA) as a promising alternative method to increase AQ production and cell growth in the cell suspension cultures of *P. multiflorum*.

### 2.6. Phenolic Compound Profiles in the Cell Suspension Culture

#### 2.6.1. Individual Phenolic Compounds

Quantitative and qualitative examinations of phenolic compounds from the PGR- (MS liquid medium containing 4% sucrose supplemented with 100 µM l-glutamine, 1 mg/L 2,4-D, and 0.5 mg/L TDZ), JA-, and SA-elicited and control cell culture extracts were performed using ultra-HPLC (Table 1). The phenolic compounds of the PGR-, JA-, and SA-elicited and control cell culture extracts were identified by comparisons of the retention time and UV spectra of authentic standards, and the quantitative data were calculated from calibration curves. The PGR-, JA-, and SA-elicited and control cell culture extracts contained flavonols and hydroxybenzoic and hydroxycinnamic acids (Table 1).

The JA-, SA-, and PGR-elicited cell cultures showed a higher amount of flavonols and hydroxybenzoic and hydroxycinnamic acids than the control cells of *P. multiflorum* (Table 1). Gallic, protocatechuic, gentisic, β-resorcylic, and syringic acid were higher in the JA, SA, and PGR cell cultures than in the control cell cultures. JA and SA enhanced the phenolic acid composition in *Vitis vinifera* suspension culture [30]. Gallic acid content was higher in the cell suspension culture of *Acer ginnala* [31] and *A. absinthium* [23]. The contents of *p*-coumaric, *p*-hydroxybenzoic, protocatechuic, salicylic, and syringic acid were higher in *Schisandra chinensis* calli [32]. SA has also been demonstrated to positively influence the biosynthetic pathway of phenolic acids [33]. The levels of ferulic, caffeic, *o*-coumaric, *p*-coumaric, *m*-coumaric, and chlorogenic acid were higher in the JA, SA, and PGR cell cultures than in the control cell cultures. Consistent with our results, caffeic acid content was higher in the SA-elicited cell cultures of *Salvia miltiorrhiza* [34]. The contents of rutin, quercetin myricetin, hesperidin, formononetin, biochanin A, kaempferol, and naringenin were higher in the JA, SA, and PGR cell cultures than in the control cell cultures. Flavonoid content was stimulated by MeJA and SA induction, which were 2.1 and 1.5 times higher when compared with the control cell suspension cultures of *Hypericum perforatum* [35]. The levels of pyrogallol, resveratrol, veratric acid, and vanillin were higher in the JA, SA, and PGR cell cultures than in the control cell cultures (Table 1). A combination of JA and l-glutamine increased resveratrol production by up to 10-fold in the cell suspension culture of *V. vinifera* [36]. JA elicited resveratrol production in the cell suspension culture of *V. vinifera* [37].

#### 2.6.2. Total Phenolic Content (TPC) and Total Flavonoid Content (TFC) in the Cell Suspension Culture

Phenylpropanoid or flavonoid biosynthetic pathways are among the most frequently observed metabolic pathways that are induced upon the addition of elicitors in plant tissue or cell cultures [10]. In the present study, the JA- and SA-elicited cell cultures could prominently induce the accumulation of total phenolics and flavonoids.

**Figure 4:**
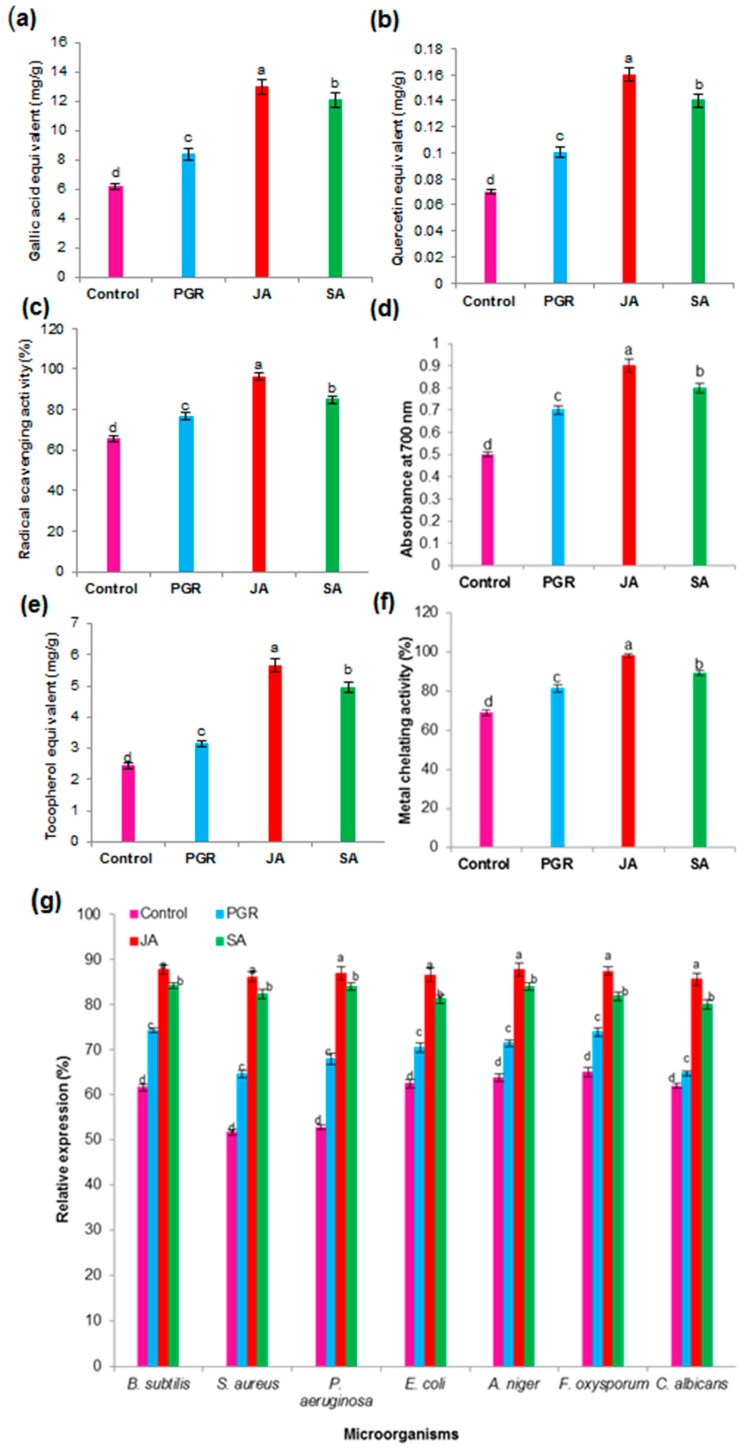
Effects of plant growth regulators (PGRs), JA and SA on levels of total phenolic and flavonoid content, antioxidant and antimicrobial activity in cell suspension cultures of *P. multiflorum* (**a**) Total phenolic content (TPC); (**b**) Total flavonoid content TFC; (**c**) Free radical-scavenging activity by 2,2-diphenyl-1-picrylhydrazyl (DPPH) method; (**d**) Reducing power; (**e**) Total antioxidant capacity by phosphomolybdenum method; (**f**) Metal chelating activity; (**g**) Antimicrobial activity. Means ± standard deviation of three replicates followed by the same letters are not significantly different according to DMRT at *p* ≤ 0.05.

Figure 4a shows that the contents of total phenolics were significantly increased by 12.95 mg/g, 12.11 mg/g, and 8.34 mg/g gallic acid equivalent (GAE) in the JA, SA, and PGR cell cultures (control, 6.15 mg/g GAE). Total flavonoid contents were higher in the cell cultures elicited with JA (0.16 mg/g quercetin equivalent (QE), SA (0.14 mg/g QE), and PGRs (0.10 mg/g QE) than in the control (0.07 mg/g QE) cell cultures (Figure 4b). Our results were consistent with those of previous studies; total phenolic and flavonoid contents were increased by JA and SA in the cell suspension culture of *P. ginseng* [38]. JA and SA enhanced phenolic and flavonoid accumulation in the suspension cultures of *A. absinthium* [39]. Cell suspension cultures enhanced the production of total phenolic and flavonoid contents in *A. absinthium* [23]. Our results suggest that JA and SA elicitation increased the level of individual and total phenolic compounds in the cell suspension cultures of *P. multiflorum*.

#### 2.6.3. Antioxidant Activity in the Cell Suspension Culture

The antioxidant potential of medicinal and food plant extracts has been attributed to the presence of phenolic acids [38]. The antioxidant capacity of PGR-, JA-, and SA-elicited and control cell cultures was evaluated using free radical scavenging, reducing potential, phosphomolybdenum assay, and metal chelating activity. Figure 4c shows that high antioxidant activity was exhibited in the cells elicited by JA (96.15%), SA (84.75%), and PGRs (76.55%) when compared with the control (65.25%) cells. The phenolic and flavonoid levels were higher in the JA, SA, and PGR culture cells, which directly influenced their antioxidant potential. Figure 4d shows the reducing capacity of *P. multiflorum* cell culture extracts, which suggests that JA, SA, and PGR culture cells have more antioxidant potential than the control cells. The antioxidant capacity of JA, SA, and PGR culture cells and control cell culture extracts was determined using the phosphomolybdenum method. The antioxidant capability of the JA, SA, and PGRs culture cells was higher than that of the control cell culture extracts (Figure 4e). The chelating agent may inhibit radical generation by stabilizing transition metals, thus reducing free radical damage. Ferrozine can form complexes with ferrous ions. When the formation of the chelating agent complex (red color) is interrupted, the red color of the compound decreases. Figure 4f shows that the percentage of metal scavenging capacity exhibited by the JA (98.05%), SA (89.15%), and PGR culture cells (81.30%) was high when compared with the control (69.15%) cells. JA- and SA-elicited suspension cells increased the antioxidant activity of *P. ginseng* [38] and *A. absinthium* [39], which is consistent with our results.

#### 2.6.4. Antimicrobial Activity in the Cell Suspension Culture

Polygonum species have excellent antimicrobial activities [40,41]. JA, SA, PGR, and control cell cultures showed variable antimicrobial activity, as revealed by the growth inhibition zones (Figure 4g). The results of the disk diffusion method demonstrated that JA, SA, PGR, and control cell culture extracts had antibacterial (Gram-positive and Gram-negative bacteria) and antifungal activities. Figure 4g shows that the JA, SA, and PGR cell culture extracts exhibited higher antibacterial and antifungal activities than the control cell extracts of *P. multiflorum*. Similarly, JA- and SA-elicited cell suspension cultures showed increased antibacterial and antifungal activities [42]. These results were compared with chloramphenicol and thymol as the positive controls for bacteria and fungi, respectively. Our study showed that JA and SA elicitation increased the level of biological activities (antioxidant and antimicrobial) in the cell suspension cultures of *P. multiflorum*. The results suggest that the cell suspension extracts of *P. multiflorum* may be used for the treatment of bacterial and fungal diseases.

#### 2.6.5. Anticancer Activity in the Cell Suspension Culture

**Figure 5:**
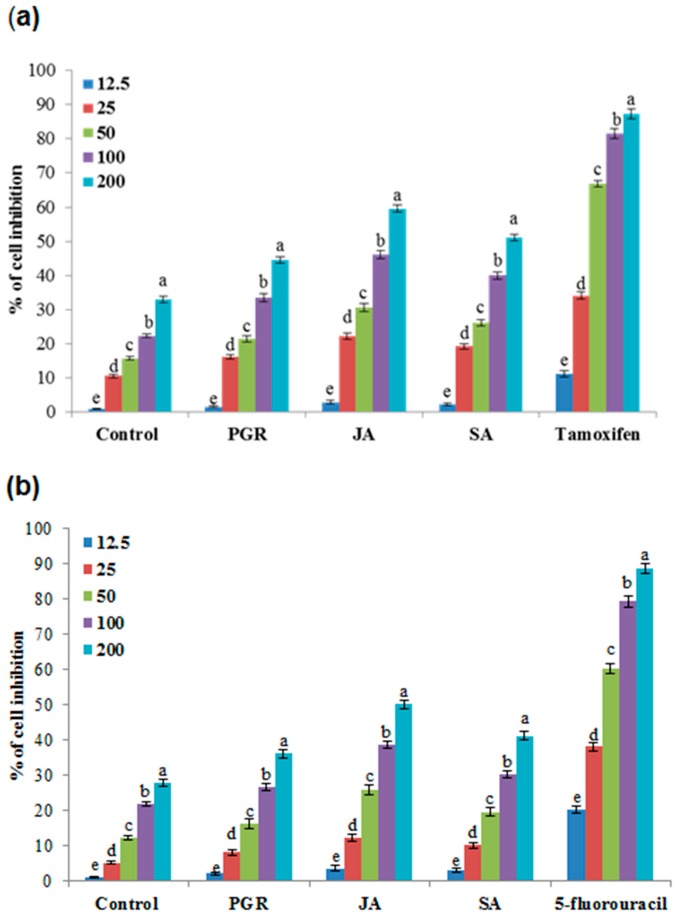
Percent cell inhibition of PGR-, JA- and SA-treated cell suspension culture extracts of *P. multiflorum* on MCF-7 and HT-29 cell lines (**a**) MCF-7; (**b**) HT-29. Means ± standard deviation of three replicates followed by the same letters are not significantly different according to DMRT at *p* ≤ 0.05.

JA, SA, PGR, and control cell cultures of *P. multiflorum* exhibited anticancer activities against the MCF-7 and HT-29 cell lines. The inhibitory effects of these extracts were compared with those of the standard tamoxifen for the MCF-7 cell line and 5-fluorouracil for the HT-29 cell line (Figure 5a,b). The cancer cell inhibition profiles were found to be concentration dependent: the higher the concentration (200 µg/mL), the greater the inhibition. MCF-7 was grown in Dulbecco’s Modified Eagle Medium (DMEM); JA, SA, and PGRs caused 59.55%, 51.12%, and 44.5%, respectively, inhibition of MCF-7 cells, whereas the control cell extracts displayed a weak inhibition of 33.02% (Figure 5a). JA, SA, and PGRs exhibited 50.09%, 41.15%, and 36.15% inhibition, respectively, of HT-29 cells, whereas the control cell extracts exhibited a weak inhibition of 28.03% (Figure 5b). On the other hand, comparison with tamoxifen (87.26%) and 5-fluorouracil (88.62%) cell line inhibition had a similarly tested dose (200 µg/mL) of MCF-7 and HT-29 respectively. MCF-7 cell line showed higher inhibition by cell extracts than HT-29 cell line (Figure 5a,b). Overall, it can be concluded that JA- and SA-elicited cell cultures may contain potential compounds that might cause the cell culture extracts to have anticancer activities.

## 3. Materials and Methods

### 3.1. Plant Materials

Seeds of *P. multiflorum* were obtained from Danong Seed Co., Ltd., Gyeonggi-do, Korea. The seeds were surface-sterilized with ethanol (70%, *v*/*v*) for 1 min and sodium hypochlorite (2%) for 10 min and then rinsed thoroughly with sterile distilled water. After sterilization, the seeds were germinated on Murashige and Skoog (MS) medium [43] with agar (0.8%, *w*/*v*) and sucrose (3%, *w*/*v*) (Sigma-Aldrich, St. Louis, MO, USA) and without any plant growth regulators (PGRs). The cultures were maintained under a 16 h photoperiod (30 μmol·m^−2^·s^−1^) provided by white fluorescent lamps at 25 ± 2 °C.

### 3.2. Establishment of Callus and Cell Suspension Cultures

Root explants from two-week-old in vitro seedlings were used as the explant source for callus initiation. The roots were cut into small segments (10–15 mm in length) and aseptically cultured on MS medium with agar (0.8%), sucrose (3%), 2,4-dichlorophenoxyacetic acid (2,4-D; 1 mg/L), 6-benzylaminopurine (BAP; 0.5 mg/L), and kinetin (KN; 0.5 mg/L) (Sigma-Aldrich, St. Louis, MO, USA). Callus cultures were incubated for three weeks in growth chambers at 25 ± 1 °C and a 16 h photoperiod (30 µmol·m^−2^·s^−1^) provided by 40 W white fluorescent lamps. Cell suspension cultures were initiated by using friable calli in liquid MS medium supplemented with 2,4-D (1 mg/L) and thidiazuron (TDZ; 0.5 mg/L; Sigma-Aldrich) in 250-mL Erlenmeyer flasks. The cultures were maintained under continuous shaking at 110 rpm in an orbital shaker and incubated at 25 ± 1 °C and a 16 h photoperiod (30 µmol·m^−2^·s^−1^) provided by cool white fluorescent lamps.

### 3.3. Effects of Auxins, Cytokinins, Amino Acid (Glutamine), Media, and Carbon (Sucrose) Sources for Biomass Accumulation and AQ Production

Fresh mass (FM; 500 mg) of the cells was cultured in liquid MS medium containing sucrose (4%) supplemented with auxins (2,4-D, naphthaleneacetic acid (NAA), and indole-3-butyric acid (IBA) (Sigma-Aldrich) and cytokinins (BAP, KN, and TDZ)) at various concentrations (0, 0.5, 1, and 2 mg/L) to regulate their effects on biomass accumulation and AQ production. MS medium devoid of PGRs was used as the control. Moreover, the effects of different concentrations (0, 25, 50, 100, and 150 µM) of l-glutamine were evaluated. The effects of various media such as MS (Murashige and Skoog), B5 (Gamborg) [27], NN (Nitsch and Nitsch) [44], and N6 (Chu) [45] were tested for the production of biomass and AQ accumulation. The effects of various concentrations of sucrose (1%, 2%, 3%, 4%, and 5%, *w*/*v*) were assessed. The cultures were harvested in duplicate (7, 14, 21, 28, and 35 days) and analyzed for biomass accumulation and AQ production. The cultures were maintained under continuous agitation at 110 rpm in an orbital shaker and incubated at 25 ± 1 °C and a 16-h photoperiod (30 µmol·m^−2^·s^−1^) provided by cool white fluorescent lamps. After 4 weeks of culture, FM, dry mass (DM), and AQ (emodin and physcion) production of the harvested cells were assessed.

### 3.4. Effects of JA and SA Elicitation for Biomass Accumulation and AQ Production

The different concentrations (0, 25, 50, 100, and 150 µM) of JA or SA (Sigma-Aldrich) were aseptically added to the culture medium containing MS salts, sucrose (4%), 2,4-D (1 mg/L), TDZ (0.5 mg/L), and l-glutamine (100 µM). The elicitors (JA and SA) were added at 25 days of cell suspension culture. The cultures were under continuous agitation at 110 rpm in an orbital shaker (25 ± 1 °C, 16-h photoperiod (30 µmol·m^−2^·s^−1^)). After 28 days of culture, FM, DM, and AQ (emodin and physcion) production of the harvested cells were evaluated.

### 3.5. Extraction and Estimation of Anthraquinones (AQs) by Using High-Performance Liquid Chromatography (HPLC)

The lyophilized cell suspensions (2 g) were finely pulverized with a mortar and pestle and extracted independently with methanol (50 mL × 3) after 20 min of sonication (Branson Ultrasonic Cleaner; Branson Cleaning Equipment Co., Shelton, CT, USA) to confirm the complete extraction of AQs. These extracts were filtered through the Advantec No. 1 filter paper (Toyo Roshi Kaisha Ltd., Tokyo, Japan), and methanol was evaporated in vacuo to dryness. The residue of the combined extract was re-dissolved in methanol and filtered through a membrane filter (0.45 μm pore size; Nalgene, New York, NY, USA). Estimation of AQs was performed using an HPLC system (Agilent 1100; Palo Alto, CA, USA) equipped with a photodiode array detector. Separation was primarily achieved using the Phenomenex C_18_ column (5 μM, 250 × 4.6 mm) (Torrance, CA, USA). The mobile phase was composed of acetonitrile (A) and 0.1% aqueous acetic acid (*v*/*v*) (B), and the following gradient program was used: linear gradient of 40%–50% A at 0–5 min; linear gradient, 50%–100% A at 5–20 min; and isocratic, 100% A at 20–25 min. A 10 min re-equilibrium was allowed between injections. The flow rate was 1 mL/min, and aliquots of 20 μL were injected into the HPLC column. Standard emodin and physcion were dissolved in methanol and diluted to different concentrations (5, 10, 25, 50, and 100 μg/mL), and 20 μL samples were subjected to HPLC three times. Standard emodin and physcion were used, and calibration plots were obtained by measuring the peak areas. Identification was performed using UV absorption spectra and retention time [40].

### 3.6. Extraction and Estimation Individual Phenolic Compounds by Using Ultra-HPLC

Lyophilized cell suspension powder (1 g) was extracted in 10 mL of acetonitrile and 2 mL of 0.1 N hydrochloric acid. The mixture was stirred for 2 h at room temperature. The extract was filtered through No. 42 Whatman filter paper (Maidstone, UK) and concentrated using a vacuum evaporator (SB-1200, EYELA, Tokyo Rikakikai Co., Ltd., Tokyo, Japan). The residue was dissolved in 10 mL of 80% aqueous methanol and filtered through a 0.45 μm membrane. The filtrate was used for ultra-HPLC (Accela UHPLC system; New York, NY, USA) with a reverse phase column (Accela, C_18_, 2.1 × 100 mm, 2.6 mm), and the absorbance was measured at 280 nm. The mobile phases were 0.1% glacial acetic acid in distilled water (solvent A) and 0.1% glacial acetic acid in acetonitrile (solvent B). The injection volume was 4 μL, and the linear gradients of the ultra-HPLC solvents were as follows: 0 min, 92% A/8% B; 0–2.2 min, 90% A/10% B; 2.2–5 min, 85% A/15% B; 5–7.5 min, 84.5% A/15.5% B; 7.5–8.5 min, 82.2% A/17.8% B; 8.5–13 min, 55% A/45% B; 13–14 min, 0% A/100% B; and 14–15 min, 92% A/8% B. The run time was 15 min, and the flow rate was 500 μL/min. Solutions of available pure known compounds—gallic acid, protocatechuic acid, pyrogallol, β-resorcylic acid, vanillic acid, caffeic acid, vanillin, *p*-coumaric acid, ferulic acid, *m*-coumaric acid, rutin, *o*-coumaric acid, chlorogenic acid, hesperedin, myricetin, resveratrol, quercetin, naringenin, kaempferol, formononetin, syringic acid, veratric acid, and biochanin A—were used as the external standards. The individual standards (25, 50, 100, and 150 µg/mL) purchased from Sigma-Aldrich were dissolved in methanol and analyzed before the samples. Phenolic compounds in the cell suspension culture extracts were identified based on the retention time and UV spectra of the authentic standards, whereas the quantitative data were calculated based on the calibration curves of the individual standards. The results were expressed as µg/g proportions of each compound that comprise the total phenolic compound content [40].

### 3.7. Estimation of Total Phenolic Content and Total Flavonoid Content

Total phenolic content (TPC) was quantified using a spectrophotometric method, according to the Folin–Ciocalteu assay [40]. Total flavonoid content (TFC) was estimated using the aluminum chloride spectrophotometric method, as described previously [40].

### 3.8. Biological Activities

#### 3.8.1. Antioxidant Activities

For antioxidant activities, 2,2-diphenyl-1-picrylhydrazyl (DPPH) free-radical-scavenging activity and reducing power were measured, and the phosphomolybdenum method and metal ion-chelating assay were performed [40,46].

#### 3.8.2. Antimicrobial Activities

The pathogenic microorganisms *Staphylococcus aureus* (KACC 10778), *Bacillus subtilis* (KACC 10111), *Pseudomonas aeruginosa* (KACC 11085), *Escherichia coli* (KACC 10495), *Candida albicans* (KACC 30062), *Aspergillus niger* (KACC 41687), and *Fusarium oxysporum* (KACC 40053) were used to test for antibacterial and antifungal activities. The pure bacterial and fungal strains were obtained from the Korean Agricultural Culture Collection (KACC), Suwon, South Korea. Antibacterial and antifungal tests were performed using the NCCLS disk diffusion method [40].

#### 3.8.3. MTT Assay

Two human cancer cell lines, namely, colon HT-29 (human colorectal adenocarcinoma) and estrogen-dependent breast MCF-7 (human breast adenocarcinoma) cancer cell lines were purchased from the KCLB (Korean Cell Line Bank, Seoul, Korea) and were used for cytotoxicity screening of the lyophilized cell suspension powder extracts (control, PGR, JA, and SA) in *P. multiflorum*. The 3-(4,5-dimethylthiazol-2-yl)-2,5-diphenyl tetrazolium bromide (MTT) colorimetric assay was performed to evaluate the cytotoxicity of the cell suspension extracts. Briefly, the cells were seeded in a flat-bottomed 96-well plate and incubated for 24 h at 37 °C and 5% CO_2_. Both cell lines were exposed to the cell suspension extract samples (control, PGR, JA, and SA). The solvent for the dimethyl sulfoxide (DMSO)-treated cells was used as the control. The cells were treated with the MTT reagent (20 µL/well) for 4 h at 37 °C, and DMSO (200 µL) was added to each well to dissolve the formazan crystals. Optical density (OD) was recorded at 492 nm by using a microplate reader. Cell viability percentage of the residual was determined as [1 − (OD of treated cells/OD of control cells)] × 100.

### 3.9. Experimental Design and Data Analysis

Each experiment was repeated three times. The data were expressed as mean ± standard deviation (SD) values. One-way analysis of variance and Duncan’s multiple range test were used to determine significant differences (*p* ≤ 0.05). All statistical tests were performed using the SPSS Ver. 20 (IBM, Chicago, IL, USA) statistical software package.

## 4. Conclusions

For the first time, we successfully established cell suspension cultures of *P. multiflorum* for the production of AQs and phenolic compounds. It can be concluded that the flask cell suspension cultures of *P. multiflorum* have the potential for scale-up studies on a commercial level by pharmaceutical industries. Higher amounts of biomass accumulation and AQ synthesis were observed in the cell suspension culture when a liquid medium with standardized concentrations of PGRs (MS + 4% sucrose + 1 mg/L 2,4-D + 0.5 mg/L TDZ + 100 µM l-glutamine) was combined with elicitors, 100 µM JA or SA. The levels of phenolic groups such as flavonols and hydroxybenzoic and hydroxycinnamic acids were higher in the JA- and SA-elicited culture cells than in the control cell cultures. The total phenolic and flavonoid contents and antioxidant, antimicrobial, and anticancer activities were higher in the JA- and SA-elicited cell cultures than in the control cell cultures. Our protocol will be useful for biochemical and bioprocess engineering and sustainable production of valuable phytochemicals (AQs and phenolic compounds) via cell suspension culture.

## Figures and Tables

**Table 1:** Ultra-high performance liquid chromatography (HPLC) analysis of the main phenolic compounds in cell suspension cultures of *P. multiflorum*.

No.	Phenolic Compounds	Concentration (µg/g DM)			
		Control	PGR	JA	SA
	Hydroxybenzoic acid				
1	Gallic acid	681.45 ± 2.0 ^d^	765.20 ± 1.5 ^c^	819.12 ± 2.0 ^a^	802.00 ± 1.5 ^b^
2	Protocatechuic acid	302.15 ± 2.5 ^d^	351.00 ± 1.0 ^c^	425.41 ± 2.2 ^a^	414.15 ± 1.0 ^b^
3	β-Resorcylic acid	172.00 ± 1.6 ^d^	235.50 ± 1.2 ^c^	354.43 ± 1.5 ^a^	340.15 ± 1.0 ^b^
4	Syringic acid	101.75 ± 1.0 ^d^	145.25 ± 1.5 ^c^	280.22 ± 1.8 ^a^	255.00 ± 2.0 ^b^
5	Gentisic acid	185.15 ± 1.5 ^d^	221.85 ± 1.0 ^c^	325.00 ± 2.0 ^a^	301.50 ± 1.5 ^b^
	Total	1442.50 ^d^	1718.80 ^c^	2204.18 ^a^	2112.80 ^b^
	Hydroxycinnamic acid				
6	Caffeic acid	165.25 ± 2.0 ^d^	211.10 ± 1.0 ^c^	265.55 ± 2.0 ^b^	285.40 ± 1.0 ^a^
7	*p*-Coumaric acid	95.00 ± 1.0 ^d^	120.00 ± 1.5 ^c^	205.10 ± 1.5 ^b^	217.32 ± 1.2 ^a^
8	Ferulic acid	312.45 ± 2.5 ^d^	345.15 ± 2.0 ^c^	415.50 ± 2.5 ^b^	441.20 ± 1.4 ^a^
9	Chlorogenic acid	72.05 ± 1.0 ^d^	105.50 ± 1.1 ^c^	211.12 ± 1.0 ^b^	215.35 ± 1.0 ^a^
10	*m*-Coumaric acid	40.00 ± 1.2 ^c^	83.16 ± 1.0 ^b^	106.00 ± 1.0 ^a^	105.10 ± 1.5 ^a^
11	*o*-Coumaric acid	102.20 ± 1.5 ^c^	145.00 ± 1.2 ^b^	202.10 ± 1.0 ^a^	202.00 ± 1.0 ^a^
	Total	786.95 ^d^	1009.91 ^c^	1405.36 ^b^	1466.37 ^a^
	Flavonols				
12	Myricetin	245.25 ± 2.0 ^c^	271.62 ± 1.5 ^b^	385.50 ± 1.2 ^a^	384.10 ± 1.5 ^a^
13	Quercetin	312.10 ± 1.5 ^d^	395.30 ± 2.1 ^c^	455.55 ± 1.0 ^a^	431.15 ± 2.0 ^b^
14	Kaempferol	105.50 ± 1.0 ^d^	152.10 ± 1.0 ^c^	215.40 ± 2.0 ^a^	201.55 ± 1.0 ^b^
15	Naringenin	82.00 ± 1.0 ^d^	95.50 ± 1.5 ^c^	145.15 ± 1.0 ^a^	120.55 ± 1.5 ^b^
16	Rutin	525.50 ± 2.0 ^d^	592.15 ± 2.5 ^c^	675.00 ± 2.0 ^a^	621.10 ± 2.5 ^b^
17	Biochanin A	112.15 ± 1.5 ^c^	125.00 ± 1.0 ^b^	162.50 ± 2.0 ^a^	160.00 ± 1.0 ^a^
18	Formononetin	121.00 ± 1.8 ^c^	145.20 ± 1.2 ^b^	180.15 ± 1.0 ^a^	180.51 ± 1.5 ^a^
19	Hesperidin	132.55 ± 2.2 ^d^	165.00 ± 1.5 ^c^	195.50 ± 1.2 ^a^	187.00 ± 1.0 ^b^
	Total	1636.05 ^d^	1941.87 ^c^	2414.75 ^a^	2285.96 ^b^
	Other Phenolic compounds				
20	Pyrogallol	925.00 ± 3.0 ^d^	1012.44 ± 2.5 ^c^	1121.15 ± 3.2 ^a^	1101.00 ± 2.0 ^b^
21	Resveratrol	212.00 ± 1.5 ^d^	305.22 ± 2.0 ^c^	414.50 ± 1.0 ^a^	400.25 ± 1.5 ^b^
22	Veratric acid	135.25 ± 1.0 ^d^	194.10 ± 1.0 ^c^	245.21 ± 1.5 ^a^	212.15 ± 1.0 ^b^
23	Vanillin	145.55 ± 1.0 ^b^	146.00 ± 1.0 ^b^	172.15 ± 1.2 ^a^	171.00 ± 2.0 ^a^
	Total	1417.80 ^d^	1657.76 ^c^	1953.01 ^a^	1884.40 ^b^

Mean ± standard deviations within a row (a–d) followed by the same letters (alphabets) are not significantly different according to DMRT at *p* ≤ 0.05.

## References

[1] Liu H., Wu W., Hou K., Chen J., Zhao Z. (2016). Deep sequencing reveals transcriptome re-programming of *Polygonum multiflorum* thunb. roots to the elicitation with methyl jasmonate. Mol. Genet. Genom..

[2] Lin L., Ni B., Lin H., Zhang M., Li X., Yin X., Qu C., Ni J. (2015). Traditional usages, botany, phytochemistry, pharmacology and toxicology of *Polygonum multiflorum* Thunb.: A review. J. Ethnopharmacol..

[3] Wang T.H., Zhang J., Qiu X.H., Bai J.Q., Gao Y.H., Xu W. (2016). Application of ultra-high-performance liquid chromatography coupled with LTQ-Orbitrap mass spectrometry for the qualitative and quantitative analysis of *Polygonum multiflorum* Thumb. and its processed products. Molecules.

[4] Malik S., Sharma N., Sharma U.K., Singh N.P., Bhushan S., Sharma M., Sinha A.K., Ahuja P.S. (2010). Qualitative and quantitative analysis of anthraquinone derivatives in rhizomes of tissue culture-raised *Rheum emodi* Wall Plants. J. Plant Physiol..

[5] Nair V.D., Panneerselvam R., Gopi R., Hong-bo S. (2013). Elicitation of pharmacologically active phenolic compounds from *Rauvolfia serpentina* Benth. Ex. Kurtz. Ind. Crops Prod..

[6] Wilson S.A., Roberts S.C. (2014). Metabolic engineering approaches for production of biochemicals in food and medicinal plants. Curr. Opin. Biotechnol..

[7] Giri L., Dhyani P., Rawat S., Bhatt I.D., Nandi S.K., Rawal R.S., Pande V. (2012). In vitro production of phenolic compounds and antioxidant activity in callus suspension cultures of *Habenaria edgeworthii*: A rare Himalayan medicinal orchid. Ind. Crops Prod..

[8] Perassolo M., Smith M.E., Giulietti A.M., Talou J.R. (2016). Synergistic effect of methyl jasmonate and cyclodextrins on anthraquinone accumulation in cell suspension cultures of *Morinda citrifolia* and *Rubia tinctorum*. Plant Cell Tissue Organ Cult..

[9] Wang W., Zhong J.J. (2002). Manipulation of ginsenoside heterogeneity in cell cultures of *Panax notoginseng* by addition of jasmonates. J. Biosci. Bioeng..

[10] Gadzovska S., Maury S., Delaunay A., Spasenoski M., Hagège D., Courtois D., Joseph C. (2013). The influence of salicylic acid elicitation of shoots, callus, and cell suspension cultures on production of naphtodianthrones and phenylpropanoids in *Hypericum perforatum* L.. Plant Cell Tissue Organ Cult..

[11] Khan M.I.R., Fatma M., Per T.S., Anjum N.A., Khan N.A. (2015). Salicylic acid-induced abiotic stress tolerance and underlying mechanisms in plants. Front. Plant Sci..

[12] Komaraiah P., Kavi Kishor P.B., Carlsson M., Magnusson K.E., Mandenius C.F. (2005). Enhancement of anthraquinone accumulation in *Morinda citrifolia* suspension cultures. Plant Sci..

[13] Shukor M.F.A., Ismail I., Zainal Z., Noor N.M. (2013). Development of a *Polygonum minus* cell suspension culture system and analysis of secondary metabolites enhanced by elicitation. Acta Physiol. Plant..

[14] Shao L., Zhao S.J., Cui T.B., Liu Z.Y., Zhao W. (2012). 2,3,5,4′-Tetrahydroxystilbene-2-*O*-β-d-glycoside biosynthesis by suspension cells cultures of *Polygonum multiflorum* Thunb and production enhancement by methyl jasmonate and salicylic acid. Molecules.

[15] Stalman M., Koskamp A.M., Luderer R., Vernooy J.H.J., Wind J.C., Wullems G.J., Croes A.F. (2003). Regulation of anthraquinone biosynthesis in cell cultures of *Morinda citrifolia*. J. Plant Physiol..

[16] Szopa A., Ekiert H. (2014). Production of biologically active phenolic acids in *Aronia melanocarpa* (Michx.) Elliott in vitro cultures cultivated on different variants of the Murashige and Skoog medium. Plant Growth Regul..

[17] Sivanandhan G., Kapil Dev G., Jeyaraj M., Rajesh M., Muthuselvam M., Selvaraj N., Manickavasagam M., Ganapathi A. (2013). A promising approach on biomass accumulation and withanolides production in cell suspension culture of *Withania somnifera* (L.) Dunal. Protoplasma.

[18] Maria L., Adam K., Daniel G. (2014). Plant growth regulators affect biosynthesis and accumulation profile of isoflavone phytoestrogens in high-productive in vitro cultures of *Genista tinctoria*. Plant Cell Tissue Organ Cult..

[19] Raj D., Kokotkiewicz A., Luczkiewicz M. (2015). Effect of plant growth regulators on the accumulation of indolizidine alkaloids in *Securinega suffruticosa* callus cultures. Plant Cell Tissue Organ Cult..

[20] Mok M.C., Gabelman W.H., Skoog F. (1976). Carotenoid synthesis in tissue cultures of *Daucus carota*. J. Am. Soc. Hortic. Sci..

[21] Murthy H.N., Lee E.J., Paek K.Y. (2014). Production of secondary metabolites from cell and organ cultures: Strategies and approaches for biomass improvement and metabolite accumulation. Plant Cell Tissue Organ Cult..

[22] Simoes-Gurgel C., Cordeiro L.D.S., de Castro T.C., Callado C.H., Albarello N., Mansur E. (2011). Establishment of anthocyanin production cell suspension cultures of *Cleome rosea* Vahl ex DC. (Capparaceae). Plant Cell Tissue Organ Cult..

[23] Ali M., Abbasi B.H., Ihsan-ul-haq A. (2013). Production of commercially important secondary metabolites and antioxidant activity in cell suspension cultures of *Artemisia absinthium* L.. Ind. Crops Prod..

[24] Nagella P., Murthy H.N. (2010). Establishment of cell suspension cultures of *Withania somnifera* for the production of withanolide A. Bioresour. Technol..

[25] Liu J.Y., Guo Z.G., Zeng Z.L. (2007). Improved accumulation of phenylethanoid glycosides by precursor feeding to suspension culture of *Cistanche salsa*. Biochem. Eng. J..

[26] Wang Y., Weathers P.J. (2007). Sugars proportionately affect artemisinin production. Plant Cell Rep..

[27] Gamborg O.L., Miller R.A., Ojima K. (1968). Nutrient requirements of suspension cultures of soybean root cells. Exp. Cell Res..

[28] Mewis I., Smetanska I.M., Müller C.T., Ulrichs C. (2011). Specific poly-phenolic compounds in cell culture of *Vitis vinifera* L. cv. Gamay Fréaux. Appl. Biochem. Biotechnol..

[29] Kang S.M., Min J.Y., Kim Y.D., Kang Y.M., Park D.J., Jung H.N., Kim S.W., Choi M.S. (2006). Effects of methyl jasmonate and salicylic acid on the production of bilobalide and ginkgolides in cell cultures of *Ginkgo biloba*. In Vitro Cell Dev. Biol. Plant.

[30] Riedel H., Akumo D.N., Thaw Saw N.M.M., Kütük O., Neubauer P., Smetanska I. (2012). Elicitation and precursor feeding influence phenolic acids composition in *Vitis vinifera* suspension culture. Afr. J. Biotechnol..

[31] Dong J., Zhan Y. (2011). Effects of several physiochemical factors on cell growth and gallic acid accumulation of *Acer ginnala* Maxim cell suspension culture. Afr. J. Biotechnol..

[32] Szopa A., Ekiert H. (2012). In vitro cultures of *Schisandra chinensis* (Turcz.) Baill. (Chinese magnolia vine)—A potential biotechnological rich source of therapeutically important phenolic acids. Appl. Biochem. Biotechnol..

[33] Cortell J.M., Halbleib M., Gallagher A.V., Righetti T.L., Kennedy J.A. (2007). Influence of vine vigor on grape (*Vitis vinifera* L. Cv. Pinot Noir) anthocyanins. 1. Anthocyanin concentration and composition in fruit. J. Agric. Food Chem..

[34] Dong J., Wan G., Liang Z. (2010). Accumulation of salicylic acid-induced phenolic compounds and raised activities of secondary metabolic and antioxidative enzymes in *Salvia miltiorrhiza* cell culture. J. Biotechnol..

[35] Wang J., Qian J., Yao L., Lu Y. (2015). Enhanced production of flavonoids by methyl jasmonate elicitation in cell suspension culture of *Hypericum perforatum*. Bioresour. Bioprocess..

[36] Vuong T.V., Franco C., Zhang W. (2014). Treatment strategies for high resveratrol induction in *Vitis vinifera* L. cell suspension culture. Biotechnol. Rep..

[37] Taurino M., Ingrosso I., D’amico L., de Domenico S., Nicoletti I., Corradini D., Santino A., Giovinazzo G. (2015). Jasmonates elicit different sets of stilbenes in *Vitis vinifera* cv. Negramaro cell cultures. SpringerPlus.

[38] Ali M.B., Hahn E.J., Paek K.Y. (2007). Methyl jasmonate and salicylic acid induced oxidative stress and accumulation of phenolics in *Panax ginseng* bioreactor root suspension cultures. Molecules.

[39] Ali M., Abbasi B.H., Ali G.S. (2015). Elicitation of antioxidant secondary metabolites with jasmonates and gibberellic acid in cell suspension cultures of *Artemisia absinthium* L.. Plant Cell Tissue Organ Cult..

[40] Thiruvengadam M., Praveen N., Kim E.H., Kim S.H., Chung I.M. (2014). Production of anthraquinones, phenolic compounds and biological activities from hairy root cultures of *Polygonum multiflorum* Thunb. Protoplasma.

[41] Salama H.M., Marraiki N. (2010). Antimicrobial activity and phytochemical analyses of *Polygonum aviculare* L. (Polygonaceae), naturally growing in Egypt. Saudi J. Biol. Sci..

[42] Abdelmohsen U.R., Ali W., Eom S.H., Hentschel U., Roitsch T. (2010). Synthesis of distinctly different sets of antimicrobial activities by elicited plant cell suspension cultures. Plant Cell Tissue Organ Cult..

[43] Murashige T., Skoog F. (1962). A revised medium for rapid growth and bioassays with tobacco tissue cultures. Physiol. Plant..

[44] Nitsch J.P., Nitsch C. (1969). Haploid plants from pollen grains. Science.

[45] Chu C.C. (1978). The N6 medium and its applications to anther culture of cereal crops. Proceedings of the Symposium Plant Tissue Culture.

[46] Su X.Q., Zhang G.J., Ma Y., Chen M., Chen S.H., Duan S.M., Wan J.Q., Hashimoto F., Lv H.P., Li J.H. (2016). Isolation, identification, and biotransformation of teadenol a from solid state fermentation of pu-erh tea and in vitro antioxidant activity. Appl. Sci..

